# A Versatile Skin-Derived Extracellular Matrix Hydrogel-Based Platform to Investigate the Function of a Mechanically Isolated Adipose Tissue Stromal Vascular Fraction

**DOI:** 10.3390/biom14121493

**Published:** 2024-11-23

**Authors:** Xue Zhang, Jan Aart M. Schipper, Rutger H. Schepers, Johan Jansma, Fred K. L. Spijkervet, Martin C. Harmsen

**Affiliations:** 1 Department of Pathology and Medical Biology, University Medical Centre Groningen, University of Groningen, Hanzeplein 1, 9713 GZ Groningen, The Netherlands; x.zhang02@umcg.nl; 2 Department of Oral and Maxillofacial Surgery, University Medical Center Groningen, University of Groningen, Hanzeplein 1, 9713 GZ Groningen, The Netherlands; j.a.m.schipper@umcg.nl (J.A.M.S.); r.h.schepers@facetofacekliniek.nl (R.H.S.); jansmaj@me.com (J.J.); f.k.l.spijkervet@umcg.nl (F.K.L.S.); 3 Department of Oral and Maxillofacial Surgery, Martini Hospital, van Swietenplein 1, 9728 NT Groningen, The Netherlands

**Keywords:** stromal vascular fraction, extracellular matrix, MMP, ECM, ECM turnover

## Abstract

*Introduction:* To accelerate cutaneous wound healing and prevent scarring, regenerative approaches such as injecting a mechanically derived tissue stromal vascular fraction (tSVF) are currently under clinical and laboratory investigations. The aim of our study was to investigate a platform to assess the interaction between skin-derived extracellular matrix (ECM) hydrogels and tSVF and their effects on their microenvironment in the first ten days of culture. *Material and Methods:* A tSVF mixed with ECM hydrogel was cultured for ten days. After 0, 3, 5, and 10 days of culture viability, histology, immunohistochemistry, gene expression, and collagen alignment and organization were assessed. *Results:* The viability analysis showed that tSVF remained viable during 10 days of culture and seemed to remain within their constitutive ECM. The fiber analysis demonstrated that collagen alignment and organization were not altered. No outgrowth of capillaries was observed in (immuno)histochemical staining. The gene expression analysis revealed that paracrine factors *TGFB1* and *VEGFA* did not change and yet were constitutively expressed. Pro-inflammatory factors *IL1B* and *IL6* were downregulated. Matrix remodeling gene *MMP1* was upregulated from day three on, while *MMP14* was upregulated at day three and ten. Interestingly, *MMP14* was downregulated at day five compared to day three while *MMP2* was downregulated after day zero. *Conclusions:* Skin-derived ECM hydrogels appear to be a versatile platform for investigating the function of a mechanically isolated adipose tissue stromal vascular fraction. In vitro tSVF remained viable for 10 days and sustained the expression of pro-regenerative factors, but is in need of additional triggers to induce vascularization or show signs of remodeling of the surrounding ECM. In the future, ECM-encapsulated tSVF may show promise for clinical administration to improve wound healing.

## 1. Introduction

Cutaneous wound healing is essential to restore its integrity and barrier function after injury [1]. Wound healing consists of regeneration and tissue repair processes involving soluble mediators, blood cells, and parenchymal cells. These can be categorized as four overlapping phases: hemostasis, inflammation, proliferation, and remodeling [2]. In wound healing, the interplay of the innate immune system (macrophages) with mesenchymal cells (fibroblasts) and vascular cells is pivotal [3]. Development and outgrowth of a vascular network into healing wounds is a ‘no-brainer’ because it warrants exchange of oxygen, carbon dioxide, nutrients, and their waste products [4]. Macrophages orchestrate much of the healing process through the secretion of paracrine factors that attract, activate, and regulate mesenchymal cells like fibroblasts [5]. Fibroblasts are the predominant professional remodelers of the extracellular matrix (ECM) and make use of the transient scaffold that is formed after a wounding lesion has formed, i.e., the fibrin clot [6]. Moreover, the fibrin clot contains a plethora of growth factors, generally released from activated platelets captured in the clot. Among these are platelet-derived growth factor (PDGF-BB) and transforming growth factor (TGF-β1) that attract and activate mesenchymal cells. This transforms resident dermal fibroblasts to myofibroblasts that act as natural ‘stitches’ to contract the wound [7]. Together, fibroblasts and myofibroblasts degrade the ‘old’ ECM while depositing new ECM in the course of wound healing [8]. The fibrin clot also contains vascular endothelial growth factor (VEGF), which, together with the oxygen gradient, promotes vascular ingrowth [9,10]. Macrophages, which enter wounds already within 24 h, remodel ECM, e.g., through the secretion of ECM-degrading proteases (matrix metalloproteinases (MMPs), a-disintegrin and metalloproteinases (ADAMs), a-disintegrin and metalloproteinase with thrombospondin motifs (ADAMTSs), and cathepsins) [11]. The regulation of healing is achieved by the secretion of various cytokines and chemokines [12].

To accelerate wound healing and prevent scarring, regenerative approaches such as injecting adipose tissue or its components are currently under clinical and laboratory investigations [13]. Adipose tissue consists of parenchymal cells, i.e., adipocytes, that are supported by the stromal vascular fraction (SVF) comprising connective tissue and vessels. Originally, the SVF was isolated by enzymatic treatment of fat to produce the cellular stromal vascular fraction (cSVF) but recently, mechanical isolation became more popular, which yields the tissue-like stromal vascular fraction (tSVF) [14]. Tissue integrity is maintained only in tSVF due to the presence of the extracellular matrix (ECM) that binds the connective tissue cells while cSVF is a suspension of cells. Cutaneous wound healing is accelerated after the administration of the patient’s own tSVF [15,16,17,18]. The dogma dictates that the phenomenon depends on the presence of adipose tissue-derived stromal cells (ASCs) in tSVF [19]. However, cultured ASCs likely differ in phenotype from their tissue resident ‘precursors’ whether these are pericytes, perivascular cells, or otherwise [20].

The extracellular matrix is essential for architectural support in tissues but also to promote cell migration and vascularization during wound healing. Our previous research showed that macrovascular human umbilical cord-derived endothelial cells (HUVECs) mixed with pericytic-like cells, i.e., ASCs, form vascular networks in tumor-derived ECM (Matrigel) but not in organ-derived hydrogels [21]. Remarkably, microvascular ECs like human microvascular ECs (HMECs) and human pulmonary MECs (HPMECs) did form networks in organ-derived ECM hydrogels independent of pericytic cells [22,23]. We recently published that in skin and lung gels, fibroblasts do augment vascular networks formed by HMEC/HPMEC by influencing ECM remodeling [24]. These results reminisce part of the working mechanism of tSVF after administration in the dermis: the interplay of ECM, developing vessels and mesenchymal cells. Our recent data show that vascular network formation demands soft(er) hydrogels while stiff(er) hydrogels inhibit. The underlying mechanosignaling comprises the surface retention of β-catenin in a focal adhesion kinase (FAK)- and Rho-associated kinase (ROCK)-regulated fashion (under review elsewhere). While this shows that the interaction between endothelial cells and ECM hydrogels supports vascularization, it is unknown whether these hydrogels also promote vascular outgrowth of encapsulated tSVF. From a different perspective, we showed that lipografting reversed dermal scars, through the long-term induction of extensive ECM remodeling and normalization. Two consecutive lipografts in mature cutaneous scars, i.e., at the beginning and after three months, showed a long-term influx of macrophages accompanied by ingrowth of vessels [15,25]. In dermal biopsies, the role of mesenchymal cells was impossible to judge due to an unfortunate lack of markers. We did, however, notice an increase in PDGF receptor β (PDGFRβ)-expressing cells, which are expectedly fibroblasts. While tSVF comprises abundant mesenchymal cells like ASCs and fibroblasts, it is warranted to better understand their role in wound and scar remodeling.

To date, adequate in vitro models to investigate the influence of tSVF in a dermal environment are lacking. Therefore, we established a platform to assess the interaction between skin-derived ECM hydrogels and mechanically derived tissue SVF and their effects on their microenvironment in early phases. We tested the hypothesis that tSVF promotes vascularization, expresses pro-regenerative factors, and remodels ECM in a skin-derived ECM hydrogel model.

## 2. Material and Methods

### 2.1. Hydrogel Generation

Hydrogels were produced according to a previously published protocol [24]. Porcine skin was purchased from a slaughterhouse (Kroon Vlees, Groningen, The Netherlands). The skin was cut into small pieces (1 cm^3^) and mixed with ice-cold Dulbecco’s phosphate-buffered saline (DPBS) (Lonza Walkersville, Inc., Walkersville, MD, USA). The mixture was minced in a regular kitchen blender with rotating blade knives (Bourgini, Breda, The Netherlands) with DPBS until it formed a homogeneous paste. The tissue homogenate was sonicated using an ultrasonic homogenizer (Sigma Aldrich, Zwijndrecht, The Netherlands) at 100% power for 1 min, collected by centrifugation, washed using DPBS twice, and incubated with 0.05% trypsin in DPBS (Thermo Fisher Scientific, Waltham, MA, USA) at 37 °C with constant shaking for 4 h. After washing twice with PBS, the slurry was incubated in ultrapure water with constant shaking at 37 °C overnight. Next, the tissue homogenate was treated with excess saturated NaCl (6 M) for 3 h. Subsequently, the homogenate was incubated in 1% SDS (Sigma-Aldrich, Zwijndrecht, The Netherlands) and 1% Triton X-100 (Sigma-Aldrich, Zwijndrecht, The Netherlands), followed by 1% sodium deoxycholate (Sigma-Aldrich, Zwijndrecht, The Netherlands) and 30 μg/mL DNase (Roche Diagnostics GmbH, Mannheim, Germany) in 1.3 mM MgSO_4_ and 2 mM CaCl_2_; Milli-Q^®^ water was used as the base solution. All incubations were under shaking at 37 °C overnight. The crude ECM was washed three times with MilliQ^®^ water between all incubations. Lastly, the homogenate was washed for one hour with DPBS under constant shaking; this was repeated six times, it was collected after centrifugation at 3,000× *g*, and 70% ethanol was added for overnight sterilization at room temperature (RT). The skin ECM samples were frozen in liquid nitrogen and lyophilized with a freeze dryer (Labconco, Kansas City, MO, USA) before being ground to a fine powder with an Ultra-Turrax homogenizer (IKA, Staufen, Germany). To generate hydrogels, 20 mg/mL of ECM powder was digested with 2 mg/mL of porcine pepsin (Sigma-Aldrich, Zwijndrecht, The Netherlands) in 0.01 M HCl with constant stirring at RT for 24 h. After digestion, the ECM was neutralized by adding 1/10th of the volume of 0.1 M NaOH and subsequently 1/10th of the volume of 10xDPBS to generate an isotonic and neutral-pH ECM pregel, which was stored at 4 °C until use.

### 2.2. Generation of Tissue-like Stromal Vascular Fraction and Mixing with Hydrogel

**Figure 1:**
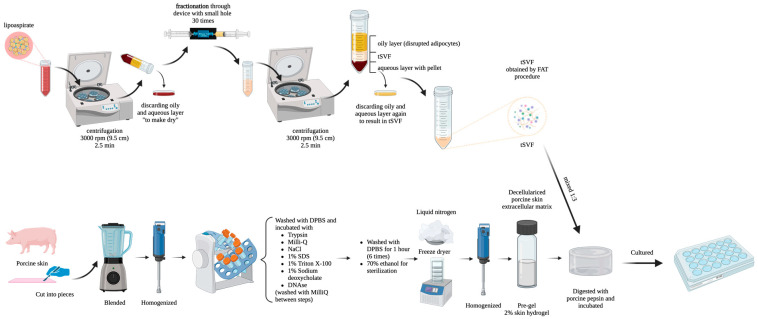
The process of the generation of tSVF and porcine skin decellularized extracellular matrix hydrogels.

Adipose tissue was obtained from anonymous donors that provided consent for use of their tissue and that underwent surgical procedures where adipose tissue was waste material to be disposed. Adipose tissue was first centrifuged, then fractionated with a 1.4 mm Tulip sizing transfer (Tulip Medical Products, San Diego, CA, USA) between 10 mL syringes (30 times) and then centrifuged again according to a previously published protocol (Figure 1) [26]. Contents of the syringes were assessed for the presence of 90% (*v*/*v*) oil after centrifugation to confirm that the majority of adipocytes were disrupted. If this quality control was not met, the sample was discarded.

### 2.3. Culture of tSVF Mixed Hydrogels

The tissue-like stromal vascular fraction was mixed with the pregel in a 1:3 *v*/*v* ratio and incubated in wells of a 48-well plate (Figure 1). After our initial experiments, we evaluated various ratios of SVF to skin-derived hydrogel, including 1:2, 1:4, and 1:8. At higher SVF content, such as a 1:2 ratio, gel formation proved challenging. In contrast, at lower SVF content, such as a 1:8 ratio, the reduced cell density hindered subsequent observations. To optimize both gel formation and cell concentration, we determined that a 1:3 ratio was the most suitable for further experiments. All experiments were repeated three times (three independent experiments). These mixtures were incubated for 10 days at 37 °C. After 0, 3, 5, and 10 days of culture, these hydrogels were fixed with paraformaldehyde and paraffin-embedded for further assessments. The culture medium comprised the endothelial culture medium, which was RPMI (Lonza, Switzerland), 20% fetal bovine serum (FBS, Gibco Life Technologies, Breda, The Netherlands), 50 µg/mL crude endothelial cell growth factor (bovine brain extract, homemade), 2 mM L-Glutamine (Sigma-Aldrich, Zwijndrecht, The Netherlands), 5 U/mL Heparin, 100 U/mL Penicillin, and 100 μg/mL Streptomycin (Sigma-Aldrich, Zwijndrecht, The Netherlands). Incubation was at 37 °C, 5% CO_2_, and 100% humidity.

### 2.4. Viability

The viability of cells at four time points (0, 3, 5, and 10 days) was determined using an inverted fluorescence microscope (EVOS model M5000, Thermo Fisher, Waltham, MA, USA) after vital staining with Hoechst (nuclei), propidium iodide (PI, dead cells), and Calcein AM (living cells). The preparation of the working solution is shown in Table 1. The medium was removed from the tSVF–hydrogel mixed samples and samples were washed twice with warm PBS (37 °C). The working solution was added to completely cover the sample and aluminum foil was applied for protection from light. Well plates were put in a shaker plate for 10 min. They were incubated at 37 °C for 20 min with 5% CO_2_. The samples were washed twice with DPBS to remove excess staining solution, and then a serum-free medium was added to maintain cell viability. The samples were imaged using the fluorescent filters shown in Table 2. The proportion of live cells was evaluated using Fiji software (v.1.54, open source on fiji.sc) by counting 50 blue nuclei and then checking the number of viable cells stained with Calcein AM (green) among them, calculating the proportion of viable cells.

### 2.5. (Immuno)histochemical Analysis

After 0, 3, 5, and 10 days of culture, the gels were fixed in 2% paraformaldehyde (PFA) for 20 min. Then, the samples were pre-embedded in 2% agarose (Invitrogen, Zwijndrecht, The Netherlands) to avoid shrinkage of the hydrogel during dehydration. After dehydration and paraffin embedding, samples were cut into 4 µm thin sections. The sections were deparaffinized and rehydrated.

For hematoxylin and eosin (H&E) staining, sections were stained in a hematoxylin solution for 10 min. Then, they were washed in tap water for 1–5 min, until the sections turned blue. After differentiating in 70% ethanol for 5 s, sections were washed for 1–5 min in tap water until a blue color appeared. Then, sections were transferred to an eosin solution for 10 min. They were rinsed in tap water for 1–5 min, then dehydrated, cleared, and mounted using a xylene-based medium.

The sections were incubated overnight with 0.1 M Tris/HCL buffer (pH 9.0) for alpha-smooth muscle actin (αSMA) and perilipin A; von Willebrand Factor (vWF) staining was preincubated with 10 mM Tris/1 mM EDTA buffer (pH 9.0) (Table 3). Primary antibodies used in this study were directed against alpha-smooth muscle actin (αSMA, 1:200, Abcam, Cambridge, UK) to stain smooth muscle cells, von Willebrand Factor (vWF, 1:200, DAKO, Glostrup, Denmark), and perilipin A (1:200, Abcam) to stain adipocytes (Table 3). Secondary antibodies used in this study were polyclonal rabbit anti-mouse for αSMA (1:100, DAKO), polyclonal swine anti-rabbit for vWF (1:100, DAKO), and polyclonal goat anti-rabbit (1:100, DAKO) for perilipin A (Table 4). The tertiary antibody used was polyclonal swine anti-rabbit (1:100, DAKO) (Table 4).

Then, endogenous peroxidase activity was blocked with 30% hydrogen peroxide in PBS for 30 min RT. Samples were washed with PBS three times and incubated with a primary antibody and 1% bovine serum albumin (BSA) in PBS for 1 h RT. One percent of human serum (HS) for αSMA and perilipin A and 1% swine serum for vWF were added to the primary antibody and 1% BSA. Negative controls were incubated without primary antibodies. Subsequently, all samples, including negative controls, were washed with PBS three times and incubated with a secondary antibody, 1% BSA, and 1% HS in PBS for 30 min RT. Only a third antibody in 1% BSA and 1% HS in PBS for 30 min was used in αSMA staining. Staining was completed with 3,30-diaminobenzidine incubation (Sigma Life Science, St. Louis, MO, USA) and hematoxylin counterstaining of nuclei. Finally, samples were mounted with an aqueous mounting agent and visualized using light microscopy (Leica Microsystems, DM, Amsterdam, The Netherlands). Masson’s Trichrome staining was performed as well after 4 µm slides were deparaffinized. Samples were counterstained with Weighert’s Iron hematoxylin for 20 min, then incubated subsequently with Biebrich Scarlet-Avid Fuchsine for 20 min, Phosphomolybdic-Phosphotungstic Acid for 12 min, Aniline blue for 7 min, and 1% acetic acid for 5 s, and washed with demi-water between incubations. Samples were mounted with a toluene solution and visualized under a light microscope (Leica Microsystems, DM, Amsterdam, The Netherlands).

### 2.6. Picrosirius Red Staining

The samples were stained in 0.1% picrosirius red for one hour (PSR; Sigma-Aldrich). After washing with 2 changes of 0.5% acetic acid and dehydration in 3 changes of 100% ethanol, the sections were cleared in xylene and mounted in a resinous medium.

### 2.7. Gene Expression Analysis

An RT-qPCR was performed to determine the gene expression of growth factors and matrix metalloproteinase and inflammatory factors at the four time points. Experiments were repeated three times independently (all data represented are from three independent samples). Total RNA was isolated following the manufacturer’s instructions provided with a NucleoSpin RNA kit (Macherey-Nagel, Düren, Germany). Later, cDNA was synthesized on 10 ng of total RNA using RevertAid Reverse Transcriptase (Thermo Scientific, Waltham, MA, USA). We added a total of 5 µL of cDNA at a concentration of 1 µg/µL to the 10 µL transcription system. Finally, qPCR was performed with a FastStart Universal SYBR Green Master for the genes (primer sequences are listed in Table 5). Reactions were carried out in 384-well PCR plates (Thermo Scientific, USA) using a Vii7 Real-Time PCR System (Applied Biosystems, Carlsbad, CA, USA). Delta Ct (ΔCt) values were calculated and normalized to *GAPDH* expression as a reference. ΔCt values were used for the comparative quantification of gene expression.

### 2.8. Image and Statistical Analyses

Fiji was used for the quantification of immunohistochemical slides [27]. Extracellular matrix organization was quantified on picrosirius red-stained sections using the TWOMBLI (after the graphical artist Cy Twombli) macro for Fiji (v1.54) [28]. TWOMBLI assesses the collagen architecture by densitometry, which generates several surrogate parameters as presented in the following (endpoints, branch points, % high-density matrix, fractal dimension, and curvatures). The TWOMBLI plugin is an innovative method to quantify fiber organization that was previously used by our group and has now already been cited by approximately 80 papers [24]. Total sections were scanned with a Hamamatsu microscopy scanner (Hamamatsu Photonics, Hamamatsu City, Japan) at 40× magnification and five random regions of interest per sample were captured for the analysis with Fiji. In our experiment, data are presented from 3 independent experiments (n = 3). All data were expressed as the mean ± standard error of the mean (SEM). Nonparametric data were analyzed with the Kruskal–Wallis test for comparisons among three groups, followed with Dunnett’s post hoc test. A *p*-value < 0.05 was considered statistically significant. Graphs and the statistical analysis were achieved using GraphPad Prism (version 10.0.0 for Windows, GraphPad Software, Boston, MA, USA).

## 3. Results

### 3.1. Viability Results

**Figure 2:**
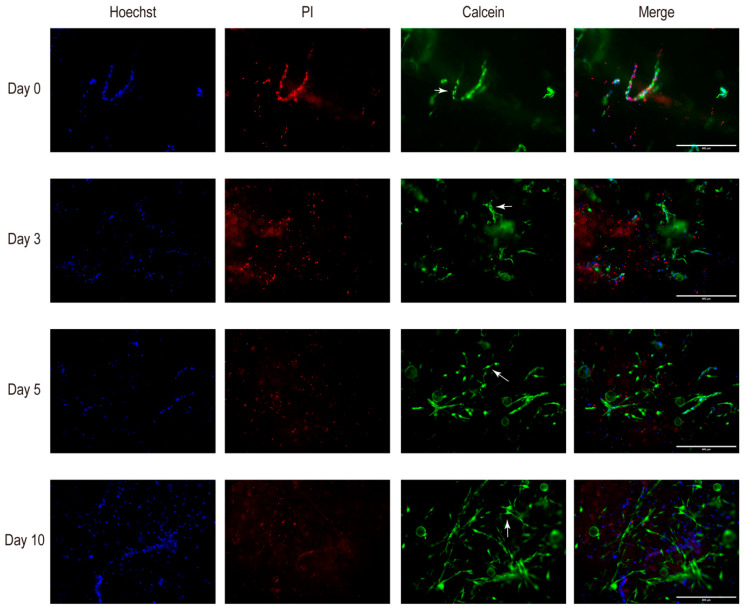
Fluorescence viability during culture of 10 days. Immunofluorescence micrographs of encapsulated tSVF in skin ECM hydrogel cultured for 0, 3, 5, and 10 days. Hoechst staining (blue) as counterstaining for DNA identifies nucleated cells. Propidium iodide staining (red) identifies dead cells. Calcein AM (green) stains viable cells. The arrows show examples of cells with a spindle-shaped appearance, reminiscent of mesenchymal cells. Original magnification: 10×, scale bar: 400 µm.

Throughout the ten-day culture period of tSVF in skin-derived ECM hydrogels, cells remained vital with negligible cell death (Figure 2). The observed proportions of dead cells at four different time points were 40%, 46%, 57%, and 52%, at days 0, 3, 5, and 10, respectively. After five days, 90% of the cells acquired a spindle-shaped appearance, reminiscent of mesenchymal cells (arrows in Figure 2).

### 3.2. Tissue SVFs Express Regeneration-Associated Genes During Culture in an ECM Hydrogel

**Figure 3:**
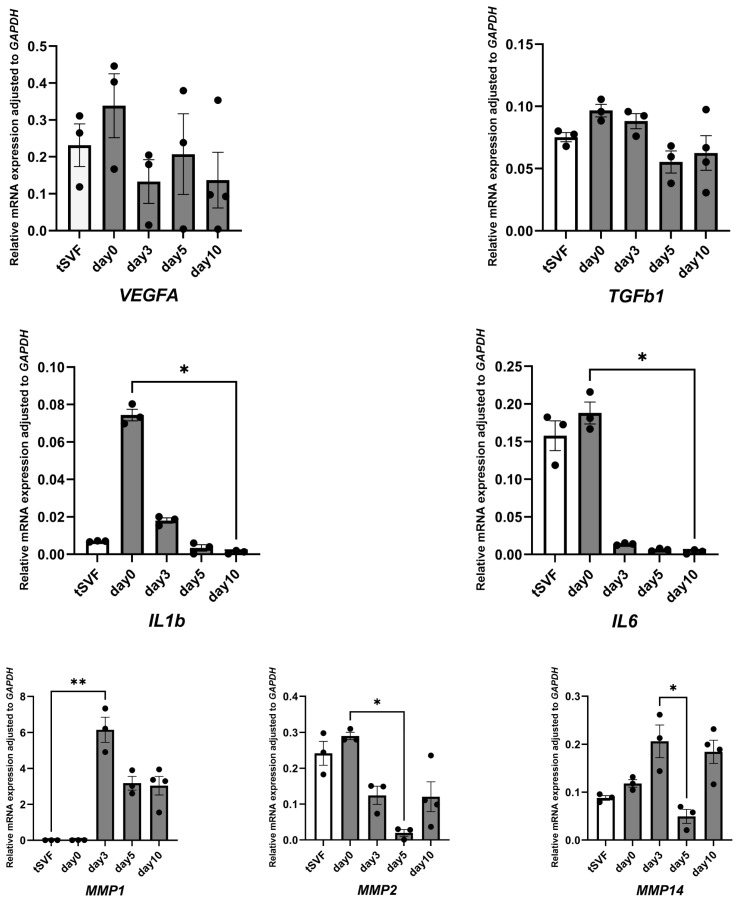
Quantitative gene expression results. Relative gene expression of wound healing-related genes in ECM-encapsulated tSVF at 0, 3, 5, and 10 days of culture. Freshly isolated tSVF served as control (tSVF). Nonparametric data were analyzed with Kruskal–Wallis test for comparisons among three groups, followed with Dunnett’s post hoc test. Data represent mean values ± SEM of three independent experiments. Each dot represents mean of three parallel samples from single patient. The data were analyzed using GraphPad Prism (v.10.2.1, GraphPad Software Inc., La Jolla, CA, USA). * *p* < 0.05, ** *p* < 0.01.

The expression of regeneration-associated, pro-inflammatory, and matrix remodeling genes in particular changed in a culture time-dependent fashion in skin-derived ECM-encapsulated tSVF (Figure 3). Paracrine factors *TGFB1* and *VEGFA* did not change and yet were constitutively expressed. Pro-inflammatory factors *IL1B* and *IL6* declined over time, with the system nearly returning to baseline or even lower levels by day 10 (*p* < 0.05). Matrix remodeling gene *MMP1* was upregulated (*p* < 0.01) compared to tSVF from day three on, while *MMP14* was upregulated at day three and ten compared to tSVF (*p* < 0.01). Interestingly, *MMP14* was downregulated at day five compared to day three (*p* < 0.05) while *MMP2* was downregulated after day zero (*p* < 0.05).

### 3.3. (Immuno)histological Results

**Figure 4:**
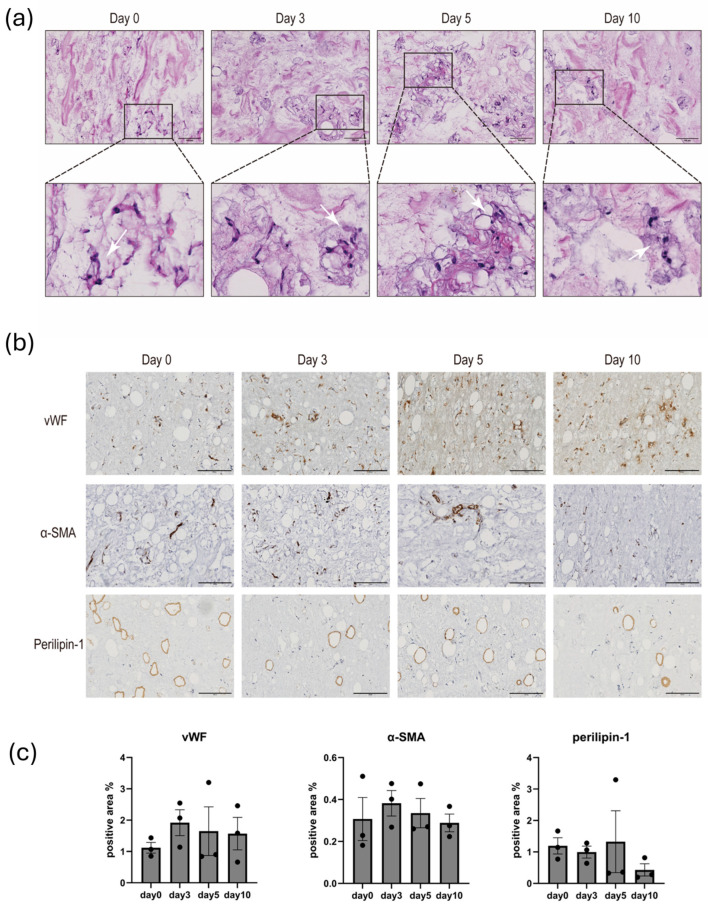
HE staining and quantitative immunohistological results. Hematoxylin–eosin-stained (**a**) and immunohistochemically stained (vWF—vasculature, αSMA—myofibroblasts, perilipin-1—adipocytes). (**b**) Micrographs and the quantification of ECM-encapsulated tSVF, cultured for 0, 3, 5, and 10 days. Calculated statistical data (**c**). Insets and arrows depict ‘islands’ of tSVF inside the skin-derived ECM hydrogels. Densitometry was used to determine the percentage of stained area (positive area). Nonparametric data were analyzed with the Kruskal–Wallis test for comparisons among three groups, followed with Dunnett’s post hoc test. Mean values are represented by bars and SEM values are represented by the scales. The data are from 3 independent experiments. Five random ROIs were measured for every single sample. Each dot represents the mean of three parallel samples from a single patient. Original magnification—40×, scale bar—200 µm.

tSVF consists of adipose tissue that is essentially devoid of adipocytes but ECM–cell connections are still intact. In histochemically stained sections, tSVF was visible as small ‘islands’ surrounded by hydrogel (arrows, Figure 4a). The ECM fibrils surrounding tSVF had a denser staining (Figure 4a) than fibrils of the skin-derived ECM hydrogel. As it appeared, cells were retained in the tSVF ‘islands’ throughout the ten-day culture. This suggests that single cells had not migrated into the ECM hydrogel. Over the ten-day culture period, no structures had appeared as reminiscent of outgrowing capillaries. The encapsulated tSVF contained residual intact adipocytes as shown by the expression of perilipin (Figure 4b). It is not possible to determine the fraction of intact adipocytes that survived mechanical fractionation; yet, in all cases, 90% of oil was formed during tSVF generation, which was the quality criterion [14,29]. There was no change in the number of endothelial cells (vWF) and smooth muscle cells or myofibroblasts (αSMA) (Figure 4c).

### 3.4. Fiber Analysis

**Figure 5:**
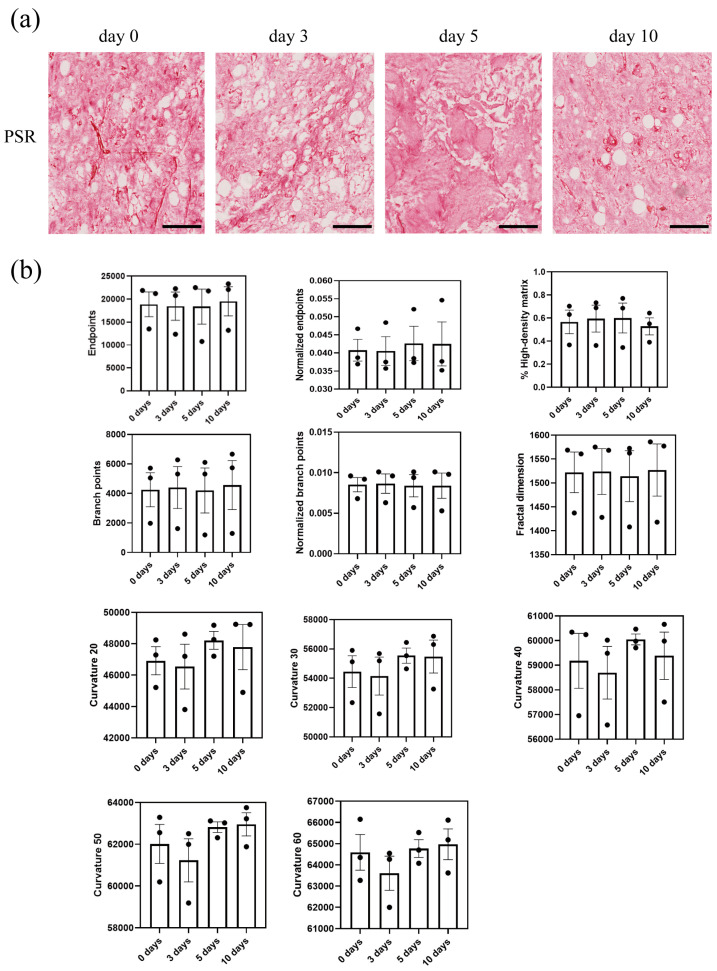
Collagen organization and alignment. Histochemical staining with picrosirius red (**a**) to visualize collagen fibers and ECM architecture of ECM hydrogel-encapsulated tSVF cultured for 0, 3, 5, and 10 days (scale bars—200 µm). Fiji plugin TWOBLI was used to assess 11 parameters (**b**) that represent surrogate descriptors of collagen matrix. Mean values are represented by bars and SEM values are represented by scales. These 11 parameters are surrogate markers for architecture and encompass fiber numbers (endpoints and normalized endpoints), apparent branching of fibers (branch points and normalized branching), shape of fibers (curvatures ’20, 30,40, 50, 60, which are arbitrary representations of the curves in fibers), and two parameters that depict the density of the collagen matrix (resp. high density matrix and fractal dimensions). None of these parameters changed over the course of ten days culture.

We analyzed the collagen organization and alignment by Fiji macro TWOMBLI, which generates several surrogate parameters (Figure 5). In general, the results showed that there was no noticeable difference between time points in the number of endpoints, branch points, % high-density matrix, fractal dimension, and curvature data. In the hydrogels, tSVF remained visible as dense ‘islands’ as shown by PSR staining (Figure 5). The high-density fibers in the tSVF did not allow for reliable analyses of collagen alignment or organization. No long-term (10 d culture) changes in fibers of the hydrogels could be identified that were associated with the presence of tSVF.

## 4. Discussion

We tested the hypothesis that tSVF promotes vascularization, expresses pro-regenerative factors, and remodels ECM in a skin-derived ECM hydrogel model. This system can function as an in vitro model for clinical tSVF injection in the patient’s skin and its interaction with the host ECM. Our main results show that tSVF lacks adequate stimulation to sprout vascular networks while skin ECM did not promote the migration of tSVF cells too. Interestingly, skin ECM induced the downregulation of pro-inflammatory genes. In contrast, matrix remodeling genes were modulated during culture, but no apparent remodeling of skin ECM was observed during the ten-day culture period. Longer culturing was not possible due to a steep decline in viability of encapsulated cells.

To the best of our knowledge, this is the first study that investigated the use of ECM hydrogels and tissue SVF [29]. We showed that decellularized porcine skin extracellular matrix (ECM) hydrogels are a suitable and versatile model to study the interactions between injected tSVF and the ECM components of skin. To understand the role of inflammation, the model can be expanded by mixing in immune cells such as monocytes and macrophages or primary dermal fibroblasts or both in future experiments. Moreover, activated platelets are attractive to add to replicate the fibrin clot that is formed immediately after acute injury of the skin [30,31,32]. The addition of ECM hydrogels to tSVF is beneficial to wound healing since ECM binds adhesion receptors that mediate cell–matrix adhesion and transduce biochemical and mechanical signals into cells [33]. ECM can also bind soluble growth factors and regulate their distribution, activation, and presentation to cells [33]. It can therefore augment wound healing by improving proliferation, immune response, and matrix remodeling [34]. Tissue SVF consists of SVF cells in their native ECM [14]. tSVF expressed pro-regenerative factors that were retained inside the tSVF, which could possibly explain why there was no migration of cells outside of their native tSVF ECM that was observed. The combined of the expression of pro-regenerative factors by both tSVF and ECM could therefore be beneficial for wound healing. Although MMP genes were upregulated during the early stages of culture (days 0–3), there was a significant decline by day 5, with a slight increase in MMP2 and MMP14 expressions observed on day 10. This could be the result of interactions between different cells in the tSVF or interactions between cells and ECM, reflecting the dynamic needs of cells for matrix remodeling at different stages. Due to the limitations of cell composition and markers, we are unable to draw definitive conclusions from these observations. At the same time, there is no matrix remodeling visible in the ECM hydrogels in the ten-day course of the experiment. This could mean that matrix remodeling is not performed to a degree that the fiber organization in ECM hydrogels became visible, or that matrix remodeling is only performed within the ECM of the tSVF itself. Because the ECM of the tSVF is very dense, the altered fiber organization of tSVF itself was not measurable by the TWOMBLI analysis.

We showed that the expression of the pro-angiogenic factors *VEGFA* and *TGFB* was sustained during culture. Although cultured SVF cells (cSVF) are known to form their own vascular networks, in this skin-derived hydrogel-based system, outgrowth of capillaries was not observed. In a mouse model in which donor cellular SVF (from fluorescent-positive mice, e.g., green fluorescent protein) mixed with Matrigel^®^ was implanted in syngeneic normal mice, donor SVF cells rapidly formed vascular networks [35]. These vascular networks did not originate from the acceptor site and it was suggested that this was the result of the dynamic disassembly–reassembly of blood endothelial cells. This study showed that the implanted SVF-derived BECs require an appropriate supporting matrix (Matrigel^®^), endogenous or supplemental vascular growth factors, and macrophages of the recipient to successfully complete the construction of a new vascular network [35]. Previous research has shown that cellular SVF induces vascularization in integrin-specific hydrogels [36,37]. However, a distinction must be made between cellular SVF and tissue SVF, since tissue SVF is already bound to its native ECM. The single cells from cSVF do not have binding places yet (contrary to tSVF in which cells are already bound to their native ECM), and therefore subsequently bound these integrin-specific hydrogels and formed vascular networks. To the best of our knowledge, vascularization from tissue SVF in hydrogels has not been shown yet, although it has been shown to stimulate angiogenesis both in vitro and in vivo [38,39]. This could be explained by the fact that tSVF needs additional triggers in vitro to persuade the vessels to grow out from the tSVF. Extra layers in this model such as macrophages that differentiate into primitive pro-angiogenic endothelial cells could promote angiogenesis [40].

In this study, we used the SVF generation of the FAT procedure [41]. It is similar to the SVF gel procedure [14,42]. Multiple in vitro and in vivo animal studies have been performed with both products [14]. However, the heterogenous nature and oil remnants of these products prove to make culturing difficult. The dogma dictates that ASCs from SVF are responsible for its regenerative properties. Since multiple cell types reside in tSVF, it is not known if a single, yet unidentified, component can be held responsible or if different components act synergistically. It is important that preclinical data on tSVF, such as those presented in the current study, become available because tSVF may have different clinical applications than cSVF or ASCs. A recent study even showed that a conditioned medium from tSVF is more beneficial for diabetic wound healing in rats than a conditioned medium from cSVF [43]. tSVF remains a promising potential therapy, but its specific clinical applications and its mechanism of action must be investigated further.

We showed that pro-inflammatory factor IL1b showed an upward trend between day 0 and later time points in cultured ECM-encapsulated tSVF, although it did not reach statistical significance. The tSVF samples were analyzed directly after tSVF generation and when tSVF was mixed with a hydrogel (i.e., day 0 sample), it was first incubated for an hour, and then samples were taken for the RT-qPCR analysis. The pro-inflammatory factors were therefore most likely expressed one hour after incubation, where in the tSVF group, the expression of these factors was not started yet. However, it is also possible that mixing tSVF and the hydrogel elicits this expression.

Our study was not without limitations. Since tSVF is variable in its cellular composition, this could increase the variability of the results. Since we evaluated only the interaction between the ECM hydrogel and tSVF, it is possible that certain cells or factors that are present in vivo are needed for certain processes, such as vascularization, to occur. Obviously, our tSVF in the skin hydrogel system falls short of the body’s normal physiology. It lacks perfusion, immune cells, and innervation to mention a few. The weakness that only specific processes can be studied like vascularization and ECM remodeling is also the strength of this system. In our lab, skin-derived ECM hydrogels have been shown to be a useful system to investigate the function of a mechanically isolated adipose tissue stromal vascular fraction as well as single mesenchymal and endothelial cells. In our in vitro system, tSVF remained viable for 10 days and sustained the expression of pro-regenerative factors. Yet, to induce the vascularization or show signs of remodeling of the surrounding ECM, additional triggers appear to be required. These might originate from, e.g., macrophages or fibroblasts or both. These can be readily mixed in the system that we established. Our current in vitro studies focus on extending the model by adding additional layers such as macrophages to further study its potential in vascularization or matrix remodeling.

## 5. Conclusions

We tested the hypothesis that tSVF embedded in skin ECM hydrogel promotes vascularization, expresses pro-regenerative factors, and remodels ECM in a skin-derived ECM hydrogel model. Unexpectedly, we conclude that tSVF in this system acts in a pro-inflammatory fashion and does not upregulate vascularization while ECM remodeling is suppressed over a ten-day culture period.

## Figures and Tables

**Table 1:** The information of the working solution.

Dye	Company	Concentration (μM)
Hoechst	Thermo Fisher Scientific	33
PI	Sigma Aldrich	3
Calcein AM	Thermo Fisher Scientific	5

**Table 2:** The fluorescent filters.

Dye	Excitation/Emission	EVOS Light Cube Filter	Filter Excitation/Emission
Hoechst	350–461 nm	DAPI (violet)	337–447 nm
PI	535–617 nm	Texas Red (red)	585–624 nm
Calcein AM	490–515 nm	GFP (green)	470–510 nm

**Table 3:** Information of first antibody.

First Antibody	Host	Company	Concentration
α-SMA	Mouse	Abcam	1:200
von Willebrand factor	Rabbit	DAKO	1:200
Anti-perilipin A	Rabbit	Abcam	1:200

**Table 4:** Information of second antibody.

Second Antibody	Host	Company	Concentration
Goat Anti-Rabbit	Goat	DAKO	1:100
Swine Anti-Rabbit	Swine	DAKO	1:100
Rabbit Anti-Mouse	Rabbit	DAKO	1:100

**Table 5:** Human primer sequences used for qRT-PCR.

Target	Forward Primer (5′→3′)	Reverse Primer (5′→3′)
*GAPDH*	AGCCACATCGCTCAGACAC	GCCCAATACGACCAAATCC
*VEGFA*	CCTGAAATGAAGGAAGAGGA	AAATAAAATGGCGAATCCAA
*TGFB1*	ACTACTACGCCAAGGAGGTCAC	TGCTTGAACTTGTCATAGATTTCG
*MMP1*	GCTAACCTTTGATGCTATAACTACGA	TTTGTGCGCATGTAGAATCTG
*MMP2*	GTTCCCCTTCTTGTTCAATG	CTTGCCATCCTTCTCAAAGT
*MMP14*	GGGTGAGGAATAACCAAGTG	CTTCCTCTCGTAGGCAGTGT
*IL1B*	AAGCTGGAATTTGAGTCTGC	ACACAAATTGCATGGTGAAG
*IL6*	AGCTCAATAAGAAGGGGCCTA	TGAGAAACCCTGGCTTAAGTAGA

## Data Availability

Data contained in this article are available upon reasonable request for scientific purposes and exclude commercial use.
